# Robotic-Assisted Colonoscopy Platform with a Magnetically-Actuated Soft-Tethered Capsule

**DOI:** 10.3390/cancers12092485

**Published:** 2020-09-02

**Authors:** Mauro Verra, Andrea Firrincieli, Marcello Chiurazzi, Andrea Mariani, Giacomo Lo Secco, Edoardo Forcignanò, Anastasios Koulaouzidis, Arianna Menciassi, Paolo Dario, Gastone Ciuti, Alberto Arezzo

**Affiliations:** 1 Department of Surgical Sciences, University of Torino, 10126 Torino, Italy; mauro.verra@unito.it (M.V.); giacomo.losecco@unito.it (G.L.S.); edoardo.forcignano@edu.unito.it (E.F.); 2 The BioRobotics Institute, Scuola Superiore Sant’Anna, 56025 Pisa, Italy; andrea.firrincieli@santannapisa.it (A.F.); marcello.chiurazzi@santannapisa.it (M.C.); andrea.mariani@santannapisa.it (A.M.); arianna.menciassi@santannapisa.it (A.M.); paolo.dario@santannapisa.it (P.D.); 3 Department of Excellence in Robotics & AI, Scuola Superiore Sant’Anna, 56025 Pisa, Italy; 4 Endoscopy Unit, The Royal Infirmary of Edinburgh, Edinburgh EH16 4SA, UK; tassos.koulaouzidis@nhslothian.scot.nhs.uk

**Keywords:** robotic colonoscopy, magnetically-actuated colonoscope, minimally invasive colonoscopy, soft-tethered capsule endoscope, comparative performance analysis

## Abstract

**Simple Summary:**

Almost 2 million human beings are newly diagnosed every year with colorectal cancer. Although easy to prevent by screening colonoscopy, this is often hampered by the perception of invasiveness of the technique. We aimed to develop a new painless colonoscopy platform consisting of an active locomotion soft-tethered capsule, offering both diagnostic and therapeutic capabilities. Capsule navigation is achieved via closed-loop interaction between two permanent magnets, enhanced by accurate localization. Ex-vivo tests showed a 100% success rate in operating channel and target approach tests. Progression of the endoscopic capsule was feasible and repeatable, and interaction forces were lower if compared to conventional colonoscopy (e.g., 1.17N vs. 4.12N). The polyp detection rates were comparable between groups (91% vs. 87%, colonoscopy and Endoo respectively). The Endoo capsule allows smoother navigation than conventional colonoscopy providing comparable features. If confirmed in clinical trials, it may represent a valuable and novel screening tool for colorectal cancer.

**Abstract:**

*Background and Aims*: Colorectal cancer (CRC) is a major cause of morbidity and mortality worldwide. Despite offering a prime paradigm for screening, CRC screening is often hampered by invasiveness. Endoo is a potentially painless colonoscopy method with an active locomotion tethered capsule offering diagnostic and therapeutic capabilities. *Materials and Methods*: The Endoo system comprises a soft-tethered capsule, which embeds a permanent magnet controlled by an external robot equipped with a second permanent magnet. Capsule navigation is achieved via closed-loop interaction between the two magnets. *Ex-vivo* tests were conducted by endoscopy experts and trainees to evaluate the basic key features, usability, and compliance in comparison with conventional colonoscopy (CC) in feasibility and pilot studies. *Results*: Endoo showed a 100% success rate in operating channel and target approach tests. Progression of the capsule was feasible and repeatable. The magnetic link was lost an average of 1.28 times per complete procedure but was restored in 100% of cases. The peak value of interaction forces was higher in the CC group than the Endoo group (4.12N vs. 1.17N). The cumulative interaction forces over time were higher in the CC group than the Endoo group between the splenic flexure and mid-transverse colon (16.53Ns vs. 1.67Ns, *p* < 0.001), as well as between the hepatic flexure and cecum (28.77Ns vs. 2.47Ns, *p* = 0.005). The polyp detection rates were comparable between groups (9.1 ± 0.9% vs. 8.7 ± 0.9%, CC and Endoo respectively, per procedure). Robotic colonoscopies were completed in 67% of the procedures performed with Endoo (53% experts and 100% trainees). *Conclusions*: Endoo allows smoother navigation than CC and possesses comparable features. Although further research is needed, magnetic capsule colonoscopy demonstrated promising results compared to CC.

## 1. Introduction

Colorectal cancer (CRC) is the third most common cancer worldwide, with almost 2 million new cases diagnosed in 2018 [1]. The number of cases has increased by about 35% over the last 6 years [2] and the related death rate represents almost half of the cases diagnosed. The highest incidence rates of CRC are reported in Europe and North America. Conventional colonoscopy (CC) [3] remains the gold standard diagnostic and screening modality [4], as it offers the option to visualize and diagnose, obtain biopsies, and treat pathologies in a single session [5]. Nonetheless, CC requires bowel preparation and is often performed under conscious or deep sedation due to patient apprehension and perceived discomfort [6]. Therefore, although CRC is a prime paradigm for screening, the efficacy of screening programs can be hampered by low adoption and participation due to the actual or perceived fears in relation to CC [7]. Until the clinical introduction of colon capsule endoscopy (CCE), computed tomography colonoscopy (CTC) was the only real imaging alternative to CC, offering the option of complete colonic evaluation with limited discomfort. However, CTC does not allow either direct colonic mucosa visualisation or intervention and treatment options. Moreover, it requires even more demanding bowel preparation and has limited sensitivity and validity in the detection of small and flat colonic polyps [8]. The latter point is CCE’s strength, however a formalized and universal cleansing regimen and external capsule control remain elusive.

The limitations of traditional endoscopes have led to the conceptualization of new robotic solutions. Some of them use electromechanical actuation (e.g., the NeoGuide endoscopy system and the Invendoscope [9]), some use electropneumatic actuation (e.g., the Aer-O-Scope system [10], the ColonoSight [11], and the Endotics system [1,2]), some use magnetic actuation (e.g., the SUPCAM [12]), while some are still under development; comprehensive literature reviews with comparative and detailed analyses are reported in [13,14,15]. The Endoo European project [16], enabled by previous works [17,18,19,20,21], was conceived to design an innovative robotic system able to reduce the invasiveness of CC. The aim was to limit discomfort and the risk of perforation due to the visceral distension and consequent stress on the bowel wall caused by the “push from the rear” technique, which is required to advance a conventional semiflexible instrument. In contrast, in the Endoo approach the capsule is “pulled from the head”, which is indeed one of the distinctive features of the presented solution. Moreover, it does not need to produce significant forces or torques through inner mechanisms, such as for electrically-actuated solutions, and relies on permanent magnets that can be customized in terms of their dimensions and shapes, but which cannot be controlled in terms of strength, if not changing the distance between magnets. This paper presents for the first time a comprehensive overview of the Endoo system and shows the results of the ex-vivo tests performed in a pre-clinical setting in order to validate the technology and lead it towards a real clinical application.

## 2. Materials and Methods

### 2.1. Endoo Robotic System

**Figure 1:**
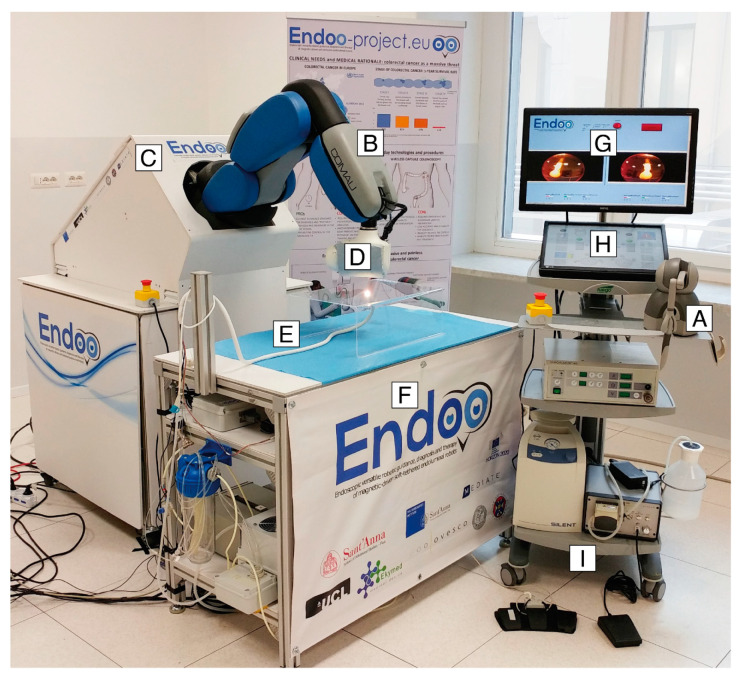
The Endoo robotic colonoscopy system: (**A**) haptic interface (joystick), (**B**) collaborative robot, (**C**) movable platform, (**D**) external permanent magnet, (**E**) capsule, (**F**) localization system, (**G**–**H**) graphical user interface, and (**I**) pumps and foot pedals. (**G**–**I**) The medical workstation.

**Figure 2:**
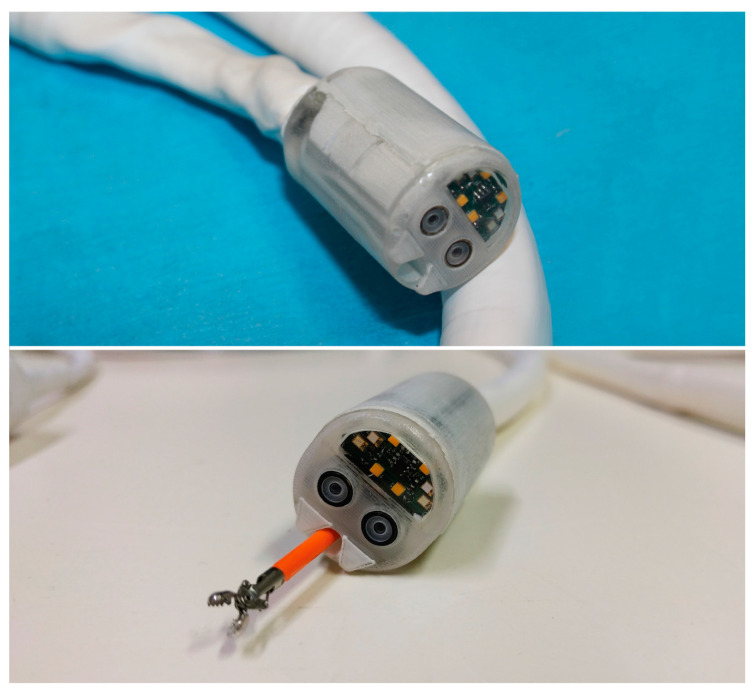
The Endoo robotic colonoscopy capsule.

**Figure 3:**
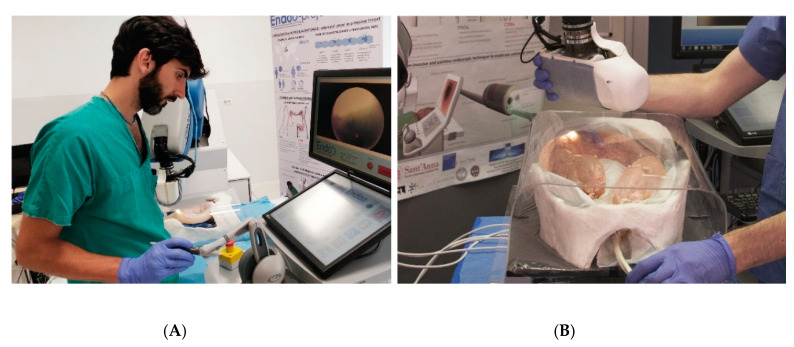
Control modalities of the Endoo platform: (**A**) teleoperated (master–slave operation via the haptic interface); (**B**) interactive (hand-guidance, operated by holding and applying pressure on the external magnet).

The Endoo system is composed of two main sub-modules, involving an external robotic driving platform (Figure 1 and Figure 2) comprising a soft-tethered capsule with diagnostic and therapeutic features (Figure 2). The robotic colonoscopy system relies on a teleoperated master–slave setup (Figure 3A) and on an interactive (Figure 3B) control architecture that assists the operator during the endoscopic procedure. Movements performed by the operator (i.e., the master) through a haptic serial control interface with 6 degrees of freedom (DoF; Geomagic TOUCH+, 3D Systems, Rock Hill, SC, USA, Figure 1A) are mapped into movements of a collaborative anthropomorphic robotic arm (i.e., the slave; COMAU Racer5-0.80 Aura, Figure 1B) mounted onto the movable platform (Figure 1C). The actuated permanent magnet, attached at the end of the robotic arm with an embedded independent DoF (Figure 1D), drives the capsule (Figure 1E and Figure 2) and controls its orientation.

The capsule houses a permanent magnet to interact with the magnetic end-effector of the robot, a tri-axial Hall effect sensor, and an accelerometer used for both localization and closed-loop control during navigation, allowing the operator to control the 5 DoF of the capsule (3 positions and 2 orientations). A force–torque sensor (Mini45 Titanium, ATI Industrial Automation, Apex, NC, USA) installed between the external magnet and the robotic end-effector monitors the interactive forces to prevent harm to the patient and to enable direct hand-guidance of the platform (Figure 3B). Thus, the endoscopist can control the capsule in a master–slave paradigm using a joystick (Figure 1A and Figure 3A) and also by direct hand-guidance of the external magnet following an interactive control modality (Figure 1D and Figure 3B). A 6-DoF magnetic localization module integrated into the operating table (Figure 1F) is used for real-time mapping of the position and orientation of the capsule during the procedure, guaranteeing the correct alignment and relative distance between external and internal magnets. A sensorized skin entirely covers the anthropomorphic robot (Figure 1B) to guarantee safe human–robot interaction, as required by the International Organization for Standardization/Technical Specifications (ISO/TS 15066:2016, i.e., a technical standard for collaborative robots) [22,23].

The robotic capsule (Figure 1E and Figure 2) guarantees all the functions of a traditional colonoscope. A soft tether, 160 cm long, is fitted to the commercial tools, which are inserted through the working channel. The front of the capsule integrates two custom-made wide-angle lenses allowing a 170° field-of-view and two 1080p complementary metal oxide semiconductor (CMOS) cameras with a 3–100 mm depth-of-focus allowing stereoscopic vision. Illumination is provided by four white light-emitting diodes (LEDs), while four green/blue UV-LEDs allow a narrow-band imaging-like functionality to enhance the visibility of vessels and other tissues on the mucosal surface. The magnetic robotic capsule and the soft tether integrate four fluidic channels: one 3.7 mm channel for suction, flushing, and tool insertion; two channels for colon insufflation and for cleaning and drying the lenses through nozzles; and one channel for irrigation of the bowel lumen (i.e., a dedicated water jet channel without restriction of the operating channel). In addition, a cable-driven actuated system (seven nylon cables measuring 0.4 mm in diameter) is integrated to allow manual variable stiffness control of the multibackbone continuum Endoo soft tether.

All functions of the Endoo capsule are actuated via a graphical user interface (Figure 1G,H), integrating monitors for real-time image streaming and connecting the different control modalities and capsule features, pumps, and foot pedals (Figure 1I) into a movable medical workstation.

### 2.2. Experimental Setting

**Figure 4:**
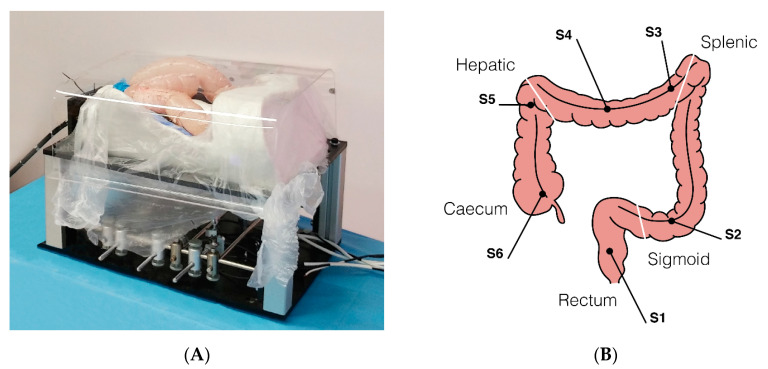
(**A**) Ex-vivo colon phantom with sensors for measuring the forces exerted on the tissue during the procedure (sensorized phantom developed by OVESCO Endoscopy AG, Tübingen, Germany). (**B**) Positions of the 6 monoaxial strain gauge sensors along the colon (S1, upper rectum; S2, mid-sigmoid tract; S3, splenic flexure; S4, mid-transverse colon; S5, hepatic flexure; S6, cecum).

A custom-made abdominal simulator (Figure 4A) was designed and specifically developed following real anatomical specifications for ex vivo tests, which was embedded in a 100–120 cm long fresh swine bowel. This was obtained through a moulding process using a real CT-reconstructed abdominal area. When the test required the measurement of forces, the bowel was connected to the simulator by means of 6 monoaxial strain gauge sensors (OMEGA LCL-005, OMEGA Engineering Inc., Karvina, Czech Republic). Sensors (S) were positioned in order to simulate the mesentery, with the aim of registering the interaction forces applied along the different colonic tracts (Figure 4B) precisely as follows: S1, upper rectum; S2, mid-sigmoid tract; S3, splenic flexure; S4, mid-transverse colon; S5, hepatic flexure; S6, cecum. In comparative tests, the operators randomly used either the Endoo system or a CC (PCF190 and Exera III, Olympus Endoscopy, Tokyo, Japan). The different experimental setups and the operators involved in the studies are schematically summarized in Table 1. The following tests reflect the ongoing experiences from the project, with different experts and trainees involved. Therefore, the tests have to be considered as a feasibility study and a pilot study at the same time. Finally, it is worth mentioning that Endoo is a “one person” technique, and when required for the different settings the pushing and pulling of the soft tether and insertion of the operating tools were performed by the same operator involved in the test.

#### 2.2.1. Operating Channel Tests

Biopsy forceps, a snare used for polypectomy, and an endoscopic needle were advanced and retracted through the operative channel up to the tip of the Endoo capsule in three different scenarios: (1) in the straight position of the soft tether, (2) after retroflection was completed, and (3) when the capsule reached the cecum. Ten expert endoscopists performed the tests. During each test and in each scenario, the success rates were derived and qualitatively evaluated by a questionnaire (1—poor; 5—excellent). Data were processed in order to extract the cumulative success rate and the mean qualitative score.

#### 2.2.2. Target Approach Tests

The endoscopists had to target a polyp in the visual field with the Endoo capsule, then (1) catch the target with the biopsy forceps, (2) touch the target with a needle, and (3) catch the target with the snare. Ten expert endoscopists performed the tests. During each test and in each scenario, success rates were derived and qualitatively evaluated to assess the stability of the manoeuvre using a questionnaire (1—poor; 5—excellent). Data were processed in order to extract the cumulative success rate and the mean qualitative score.

#### 2.2.3. Lumen Progression Tests

While the endoscopist was driving the capsule from the sigmoid tract to the splenic flexure and back (about 200 mm distance), the following were measured: (1) the time taken to conclude the path, (2) the endoscopist’s capability to control the capsule movement (translation and tilting), (3) the number of magnetic losses, and (4) the percentage of magnetic losses in which the magnetic link was restored. Ten expert endoscopists performed the tests, and during each test navigation was also qualitatively evaluated by a questionnaire (1—poor; 5—excellent). Data were processed in order to extract the average procedural time (mean ± standard deviation—SD), the total number of magnetic losses, the cumulative success rate for completing the path and restoring the magnetic link, and the mean qualitative scores for translation and tilting capabilities.

#### 2.2.4. Interaction Force Tests

Using the colon simulator equipped with force sensors, the endoscopist had to drive the capsule from the rectum to the cecum, then repeat the same task with a conventional colonoscope. Ten expert endoscopists performed the tests. Force sensor data were monitored online and recorded for post-process evaluation. Interaction force curves, ranges, and mean and cumulative forces over time were calculated for both the Endoo capsule and the CC. These parameters were grouped by the following bowel segments and compared as: S1, upper rectum; S2 mid-sigmoid colon; S3 and S4, splenic flexure and mid-transverse colon; S5 and S6, hepatic flexure and cecum. Cumulative forces over time are the data that numerically best represent the interaction and pain generated during the procedure from a physical point of view; in fact, these factors are strictly connected to momentum (mass and speed) and impulses exerted on the tissues during navigation. A non-parametric test (Wilcoxon rank sum test) was used to assess statistically significant differences between the Endoo and the CC.

#### 2.2.5. Polyps Detection Tests

Ten endoluminal lesions (i.e., artificial polyps) measuring 5–10 mm in diameter were created by stitching along each colonic model at specific sites unknown by the endoscopists: two in the sigmoid tract (at 6 and 12 o’clock), two in the descending colon (at 3 and 9 o’clock), two in the splenic flexure (at 6 and 12 o’clock), two in the hepatic flexure (at 3 and 9 o’clock), and two in the cecum (at 6 and 12 o’clock). The endoscopist had to drive the capsule from the rectum to the cecum and repeat the test with a conventional colonoscope (colon length: 100–120 cm). In both cases, the endoscopist had to report each visualized target in order to calculate the total and mean number of detected polyps and the overall rate. Ten clinicians (5 endoscopists and 5 trainees) performed the tests and a subgroup analysis was performed with trainees (colonoscope vs. Endoo), who were not biased by previous experience.

#### 2.2.6. Colonoscopy Simulation Tests

The endoscopist had to drive the capsule from the rectum to the cecum and back, then repeat the same task with a conventional colonoscope. During the tests, the following factors were measured: (1) the time taken to complete the task, (2) the distance from the anal verge in case of no completion of the test, (3) the number of magnetic losses, (3) the number of magnetic losses in which the platform was not able to restore magnetic link, (4) the number of abnormal responses of the Endoo system (e.g., tilting was no longer possible because of the relative position of the robot), and (5) the rate of loss of connection when moving back and straightening the wire. Among fifteen expert endoscopists and eight trainees, fifteen experts and six trainees were involved in testing the Endoo platform, whereas five experts and eight trainees were involved in performing a CC (overall, a total number of thirty-four procedures). Experts and trainees received a training session lasting about 15 min to explain and test the main features of the Endoo system. Data were processed in order to extract the success rates, average times, average number of magnetic losses, and success rates in restoring magnetic link.

## 3. Results

### 3.1. Operating Channel Tests

The success rate was 100% when polypectomy snares, biopsy forceps, and needles were advanced in each scenario. The mean qualitative rate of all the manoeuvres was 3/5.

### 3.2. Target Approach Tests

The success rate was 100% when polypectomy snares, biopsy forceps, and needles were advanced in every scenario. The mean qualitative rate for stability during the manoeuvres was 4.8/5.

### 3.3. Lumen Progression Tests

The success rate was 100%, with an average time of 4:06 ± 1:12 (range 2:35–6:45) minutes. The translation was judged as at least good (score: 3) by all ten experts, while six highlighted the need to aid the tether by pushing it from outside. Tilting was judged as at least good (score: 3) by all ten experts, but one found difficult to control it on the right side. The magnetic link was lost 5 times during the 10 procedures, but it was restored in 100% of the cases.

### 3.4. Interaction Forces Tests

**Figure 5:**
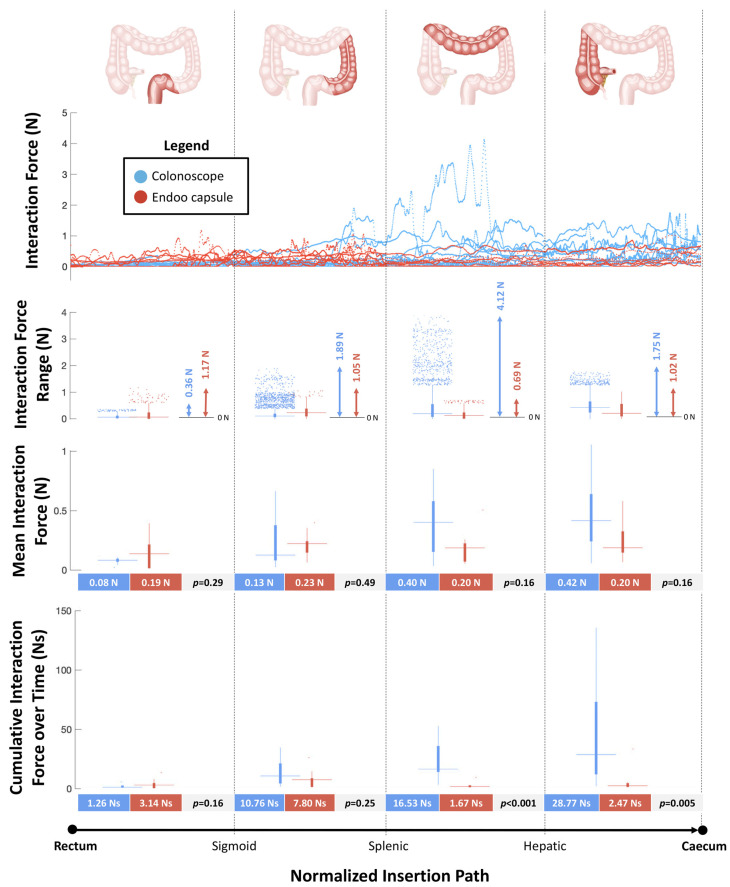
Interaction forces between the scopes (colonoscope and Endoo) and the anatomical structures (maximum output across six force sensors) as functions of the normalized insertion path from the rectum to the cecum. In each boxplot, the horizontal line displays the data median, while the vertical bars stand for data variability (25th and 75th percentiles, bold and thin whiskers, respectively) and the dots depict outliers.

Figure 5 reports the forces measured by the sensorized platform during navigation from the rectum to the cecum. The peak values of interaction forces were higher in the CC group compared with the Endoo system at the mid-sigmoid colon (S2) (1.89N vs. 1.05N), at the splenic flexure (S3) and mid-transverse colon (S4) (4.12N vs. 0.69N), and at the hepatic flexure (S5) and cecum (S6) (1.75N vs. 1.02N). The mean interaction forces were similar between the two groups in each bowel tract analysed. The cumulative interaction forces over time were higher in the CC group compared with the Endoo group at the splenic flexure (S3) and mid-transverse colon (S4) (16.53Ns vs. 1.67Ns, *p* < 0.001) and at the hepatic flexure (S5) and cecum (S6) (28.77Ns and 2.47Ns, *p* = 0.005).

### 3.5. Polyps Detection Tests

Overall, 91/100 polyps were detected by CC and 87/100 by the Endoo system (*p* = 0.16). The mean number of polyps detected per procedure was 9.1 ± 0.9 in the CC group and 8.7 ± 0.9 in the Endoo capsule group. The subgroup analysis showed that trainees were able to identify 43/50 polyps using CC and 44/50 using the Endoo system (*p* = 0.84).

### 3.6. Colonoscopy Simulation Tests

**Figure 6:**
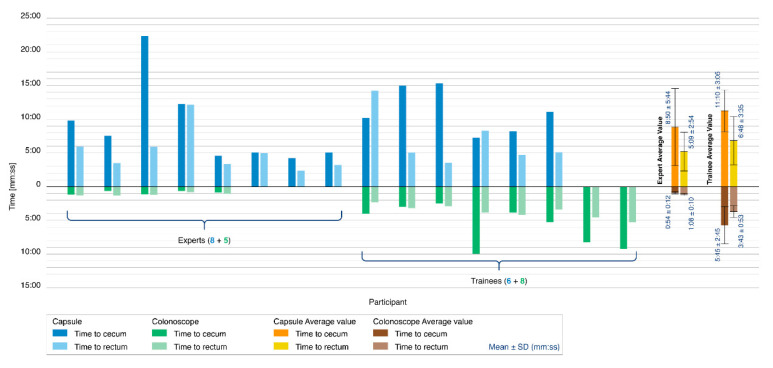
Colonoscopy simulation tests. The histogram reports the comparison between the Endoo system (blue) and the conventional colonoscope (green) in terms of the time taken to reach the cecum (dark blue/green) and time taken to reach the rectum (light blue/green). The tester populations were composed of 5 experts and 8 trainees for the conventional colonoscope and 8 experts and 6 trainees for the Endoo system. Mean times are reported on the right side of the graph.

Figure 6 reports the results of the colonoscopy simulation tests. A colonoscopy was completed in 14 of the 21 procedures performed with the Endoo system (67%). Only 8 of the 15 experts were capable of completing the colonoscopy with the Endoo platform (53%). Seven procedures were aborted because of difficulties in overcoming the hepatic flexure. All 6 trainees performed the procedures successfully (100%). When the colonoscopy was performed with a standard colonoscope, the success rate was 100% for all 13 procedures (5 experts and 8 trainees).

The mean times to complete the colonoscopy with the Endoo system, when successful, were 9:50 ± 4:55 min (from rectum to cecum) and 5:51 ± 3:19 min (from cecum to rectum), for a mean global time of 15:42 ± 6:32 min. The mean times to complete colonoscopy with the conventional colonoscope were 3:53 ± 3:12 min (from rectum to cecum) and 2:43 ± 1:26 min (from cecum to rectum), for a mean global time of 6:37 ± 4:31 min. Experts in endoscopy performed complete colonoscopies with mean times of 14:00 ± 7:37 min and 2:03 ± 0:19 min with the Endoo system and the CC, respectively, whereas the mean time for trainees were 17:58 ± 3:40 min and 9:28 ± 3:27 min with the Endoo system and the CC, respectively.

The magnetic link was lost an average of 1.28 times per complete procedure (experts: 1.25; trainees: 1.33), but it was restored in 100% of cases. External manual pushing (with the variable stiffness system activated) was requested and used by subjects in 6 of the 21 procedures (29%), and in 5 of these 6 procedures (83%) when the capsule was approaching the hepatic flexure.

## 4. Discussion

The Endoo system is the result of a long-term interdisciplinary collaboration of engineers and endoscopists, which is focused on clinical applications [19] and was largely developed during a European project within the Horizon 2020 framework (G.A.: 688592, ICT-24-2015 Robotics; Video S1 in Supplementary Material) [16]. The conception of Endoo was dictated by the clinical need for a painless robotic colonoscopy platform with capabilities similar to CC. The preliminary tests presented herein were performed to evaluate the overall Endoo system performance results in comparison with CC in ex-vivo conditions. A key objective of the Endoo system was to maintain all the features of CC in a completely new design with reduced invasiveness; the Endoo system demonstrated that the use of the 3.7 mm (internal diameter) working channel was rated as good and that the insertion of accessories, such as biopsy forceps, needles, snares, and fluid was successful, even when the tether was subjected to tight bending and capsule retroflexion.

To date, several technological solutions have been proposed to replace conventional colonoscopy. Snake-like and continuum robots have shown promise in the field of surgery. Due to their superior dexterity, they outperform the current straight-line laparoscopic tools. Degani et al. and Kwok et al. created miniaturized snake-like robots that can be inserted through narrow openings, penetrating into a patient’s chest or abdomen to conduct theranostic tasks. Taking inspiration from biology, these snake-like robots are constructed from a number of rigid components connected to each other by a flexible interface and are actuated by tendons (i.e., Highly Articulated Robotic Probe—HARP) or electric motors (i.e., i-Snake) [24,25]. Continuum robots with one or more flexible backbones have also been developed for minimally invasive surgery (MIS). Theoretically, these can achieve an infinite number of configurations, with examples by Ding et al. [26] and Xu et al. [27]. Although snake-like and continuum robots show potential for endoscopy, they are limited in their operational range to about 300 mm due to wiring and piping problems. Furthermore, these robots are “pushed” forward by translational actuators, potentially causing injury to tissue because of sliding friction between the robots and the surrounding environment. Alternatives to snake-like and continuum robots are robotic capsules. They are provided with active locomotion capabilities and have been designed and actuated by means of: (1) propellers, wirelessly controlled to guarantee 3D navigation of the capsule in a water-filled stomach [28]; (2) flagellar- or flap-based swimming mechanisms [29]; or (3) water jets, provided by a multichannel external water distribution system [30]. Robotic capsules can also feature an external locomotion approach, which usually involves the use of permanent magnets or electromagnets and entails external field sources that interact with internal magnetic components to provide navigation and steering. The benefit of the external locomotion approach with internal permanent magnets is the reduced complexity—there are no on-board actuators, mechanisms, and batteries thanks to the small integrated magnetic field source. In contrast, internal locomotion comes with the dramatic drawback of excessive internal encumbrance due to the use of actuators, transmission mechanisms, and powering modules.

The declared aim of the Endoo project was to develop a disposable system to replace CC by offering limited discomfort and reduced, if not negligible, risk of bowel perforation. In fact, CC is still considered to pose risks in terms of discomfort and perforation and is perceived as unpleasant. This is mainly due to visceral distension and consequent stress on the bowel wall created by the “push from the rear” technique, which is required to advance a semiflexible instrument. Our approach is based on an innovative idea involving an endoscope (i.e., a soft-tethered magnetic capsule) being “pulled from the head”. Moreover, this solution does not produce significant forces or torque, as it relies on the magnetic forces generated by an external permanent magnet that can be customized in terms of dimensions and shape, which is accurately and reliably controlled by a robotic platform. The possible disadvantages of this solution are reported as follows: (1) the capsule is always sliding along the colon perpendicularly to the direction of the external–internal magnetic attraction and is not really floating in the middle of the bowel lumen, which may prevent an immediate approach of the target area, possibly requiring the configuration of the external magnetic source to be changed or the patient to be rotated along the longitudinal axis; and (2) the possibility of the capsule being dropped when exceeding the range of influence of the magnetic field generated by the external permanent magnet, although this can be mitigated by the integration of a closed-loop, localization-assisted locomotion strategy.

It is worth mentioning that the Endoo magnetically actuated robotic platform ensures higher stability during operative manoeuvres than the flexible CC, as reported by the qualitative evaluation tests. Moreover, the Endoo platform showed good and reproducible progression capabilities according to qualitative and quantitative evaluation tests. In general, the capability of controlling the tilting of the capsule is good and reproducible; however, the clinicians reported the need to improve the control of the yaw angle of the soft-tethered capsule. The forward–backward navigation control is satisfactory; however, to keep the magnetic force within a safe range, the strength used in pulling the capsule may be insufficient to overcome the friction of the tether. Therefore, it was necessary to help the advancement of the Endoo capsule by externally assisting the tether with just enough force to reduce the need for the magnetic propulsion, which is then sufficient to proceed with the current platform. Data regarding the forces exerted on the colonic wall confirmed that magnetic guidance may enable a swift procedure with lower stretching forces, primarily on the proximal bowel tracts, as compared to the flexible CC. In turn, this should lead to higher comfort and to higher compliance for patients.

When combining the experimental results of both expert endoscopists and trainees in ex-vivo tests, the Endoo system showed some difficulties to complete colonoscopy compared to CC. However, considering the experiments performed only by trainees, the success rate of Endoo was 100%; this demonstrates the ease with which non-expert but “digital-native” users can become accustomed to this novel endoscopic robotic platform and makes it reasonable to envisage that training and extended use can standardize performance among users, becoming comparable to flexible CC in terms of success rate and procedure time. Furthermore, feedback from the expert endoscopists and clinicians’ subgroup revealed that some of the versed users experienced a progressive loss of manoeuvrability as the capsule moved into the proximal colon, leading in some cases to failure to complete the procedure. In particular, after the hepatic flexure, manoeuvrability (i.e., tilt and progression) became counterintuitive. Indeed, when cecum intubation was not possible, the hepatic flexure was always the endpoint of the procedure. It is, therefore, implied that the complexity in manoeuvring the capsule in the right colon depends on the tether’s relative friction, which significantly increases with the level of colonic convolution and the number of colonic curves that are overtaken. The variable stiffness control and external manual advance of the Endoo tether are the likely solutions to this. Although it was demonstrated that it is possible to navigate the Endoo capsule into the cecum, further research on materials that can cover and constitute the tether are required to increase the flexibility and reduce the resistive navigation forces.

Our experiments also demonstrate that Endoo can be an accurate and reliable platform for colonic detection of polyps, with a rate close to 100% after proper training and extended use of the platform. This, together with the effective stability of the system, makes Endoo a promising device for diagnostic and therapeutic colonoscopy of the bowel. In addition, despite the comparable diagnostic accuracy, the procedural time was still higher with the Endoo system (about two times higher). However, considering the ~1:5 procedural time ratio for CC between experts and trainees, we envisage that after proper training and extended use (shortened, as with the learning curve, by the robotic-aided technologies), the procedural time and the overall procedure success rate with the Endoo system could become comparable to those of CC.

## 5. Conclusions

In summary, we demonstrated through feasibility and pilot studies in a pre-clinical setting that the manoeuvrability of the Endoo system is comparable to CC when capsule movements were not hampered by the tether. Therefore, further efforts should focus on improving the tether features to reduce friction. Although tests were performed only in ex-vivo models, the Endoo system allowed one of the first complete colonoscopies with a soft-tethered, magnetically actuated robotic capsule. The Endoo system showed comparable accuracy in the detection of endoluminal lesions to that of CC. The time is now ideal to perform extensive comparative tests on cadavers, leading towards application in healthy humans.

Endoo’s features may make soft-tethered magnetic capsule endoscopy a candidate as a potential competitor to conventional flexible endoscopy. Clinical trials on humans will need to confirm whether a more comfortable colonoscopy procedure can be performed with comparable accuracy, time and operative skills. In context, the Endoo robotic technology is suitable for semiautonomous navigation thanks to AI-based lumen and polyps detection algorithms.

## Figures and Tables

**Table 1:** Tests Used to Evaluate the Endoo System Functionality, Pre-Compliance, Usability, and Compliance in Comparison with CC.

Tests Category	Tests ID	Platforms	Experimental Condition	Sensorized Simulator	Participants (Expert)	Participants (Trainees)	Outcomes
**Endoo basic key functionalities**	Operating Channel	Endoo	*Ex-vivo*	NO	10	0	Success rate (%) and qualitative score (1–5)
	Target Approach	Endoo	*Ex-vivo*	NO	10	0	Success rate (%) and qualitative score (1–5)
	Lumen Progression	Endoo	*Ex-vivo*	NO	10	0	Success rate (%), time (mm:ss), qualitative scores for navigation (1–5), number of capsule losses (#), and success rate for restoring link (%)
**Comparative pre-compliance**	Interaction Forces	Endoo vs. Colonoscopy	*Ex-vivo*	YES	5	5	Interaction force (IF), IF range, mean force (N), and cumulative interaction force overtime (Ns)
	Polyps Detection	Endoo vs. Colonoscopy	*Ex-vivo*	NO	5	5	Total and mean numbers of detected polyps (#) and overall rate (#/total)
**Usability and comparative compliance**	Colonoscopic Simulation	Endoo vs. Colonoscopy	*Ex-vivo*	NO	15 (15 procedures with Endoo—5 procedures with colonoscopes)	8 (6 procedures with Endoo—8 procedures with colonoscopes)	Procedural times to cecum/rectum (mm:ss), success rates (%), number of capsule losses (#), success rate for restoring link (%)

# number of occurrences.

## Supplementary Materials

Video S1—Rendering and real procedure with the Endoo system: https://youtu.be/j9Bn3WRhRZk.

## Abbreviations

CRC	Colorectal cancer
CC	Conventional Colonoscopy
CCE	Colon Capsule Endoscopy
CTC	Computed Tomography Colonoscopy
DoF	Degrees of Freedom
MIS	Minimally Invasive Surgery
CMOS	Complementary Metal Oxide Semiconductor
LEDs	Light Emitting Diodes
SD	Standard Deviation
